# Illuminating the Role of Vitamin A in Skin Innate Immunity and the Skin Microbiome: A Narrative Review

**DOI:** 10.3390/nu13020302

**Published:** 2021-01-21

**Authors:** Fritzlaine C. Roche, Tamia A. Harris-Tryon

**Affiliations:** 1 Department of Dermatology, University of Texas Southwestern Medical Center, Dallas, TX 75390, USA; fritzlaine_roche@urmc.rochester.edu; 2 Department of Dermatology, University of Rochester School of Medicine & Dentistry, Rochester, NY 14642, USA; 3 Department of Immunology, University of Texas Southwestern Medical Center, Dallas, TX 75390, USA

**Keywords:** vitamin A, retinoic acid, skin immunity, innate immunity, skin microbiome

## Abstract

Vitamin A is a fat-soluble vitamin that plays an important role in skin immunity. Deficiencies in Vitamin A have been linked to impaired immune response and increased susceptibility to skin infections and inflammatory skin disease. This narrative review summarizes recent primary evidence that elucidates the role of vitamin A and its derivatives on innate immune regulators through mechanisms that promote skin immunity and sustain the skin microbiome.

## 1. Introduction

The skin is the largest epithelial organ and acts as an essential barrier between internal organs and the outer microbial world [1]. The skin functions as a barrier against diverse microbial communities through a variety of immune defense strategies. These defense strategies are in part mediated by nutrients that influence the gene expression of anti-microbial proteins and molecules that enhance skin immunity [2]. There is increasing evidence in the literature of the essential role of vitamin A and its derivatives in skin immunity. In this review, we survey current knowledge on the role of vitamin A and its metabolite, retinoic acid, in skin innate immunity and the skin microbiome through examining primary animal and human studies. Scientific studies published between 2005 and 2020 in the databases of PubMed and Google Scholar were identified using the specific search terms ‘’vitamin A and skin innate immunity’’, ‘’retinoic acid and skin innate immunity’’, ‘’vitamin A and skin microbiome’’, ‘’retinoic acid and skin microbiome’’ and ‘’vitamin A and skin inflammation’’. A few key scientific studies published prior to 2005 were also included to provide a historical context to the evolving role of vitamin A on skin innate immunity and the skin microbiome.

## 2. Vitamin A, Retinoids, and the Skin’s Innate Immune Response

**Figure 1:**
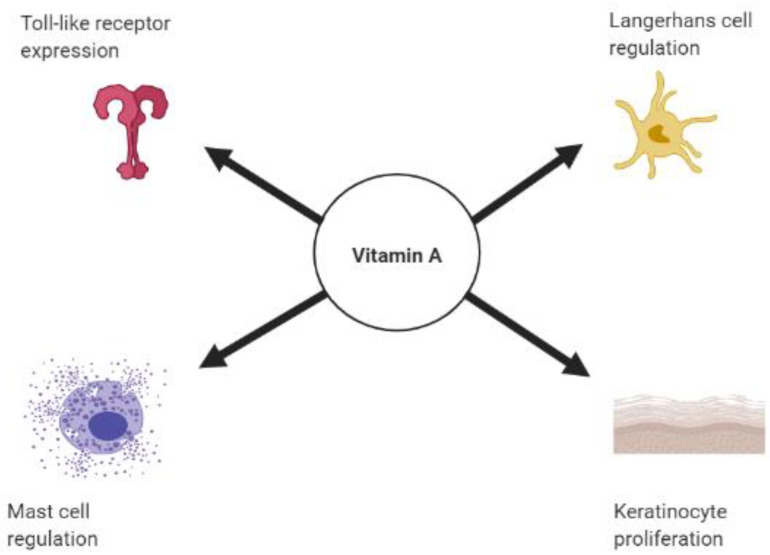
Role of vitamin A on skin innate immunity. Schematic diagram of the role of vitamin A on promoting toll-like receptor expression, mast cells, and Langerhans cell regulation and keratinocyte proliferation. Created with BioRender.

Vitamin A is a lipid-soluble essential nutrient of the diet that modulates the immune response and maintains homeostasis of epithelial tissues and mucosa through its metabolite, retinoic acid (RA) [3]. RA controls gene expression through retinoic acid receptors (RARs), which are transcription factors expressed by the skin that mediate expression of target genes [4,5]. Deficiencies in vitamin A have been linked to an increased susceptibility to skin infection and inflammation [6,7]. This susceptibility has suggested an integral role for vitamin A in promoting immune function in the skin (Figure 1).

### 2.1. Vitamin A and Toll Like Receptors

Key components of the innate immune system in skin are toll-like receptors (TLRs) [8], which are expressed in all innate immune cells, including antigen presenting cells (APCs), epithelial cells and endothelial cells [9]. Each TLR is a pathogen recognition receptor (PRR) that responds to signals from pathogens or damaged cells and induces a pro-inflammatory immune response, through the translocation of nuclear factor-kappa B (NF-κB) into the nucleus [10]. TLRs are subdivided based on their cellular localization. TLR1, TLR2, TLR4, TLR5, TLR6 and TLR11 are expressed on the plasma membrane and recognize microbial membrane lipids, lipoproteins and proteins [9]. The other group of TLRs, consisting of TLR3, TLR7, TLR8 and TLR9, are expressed on intracellular vesicles and recognize microbial nucleic acids [9].

**Figure 2:**
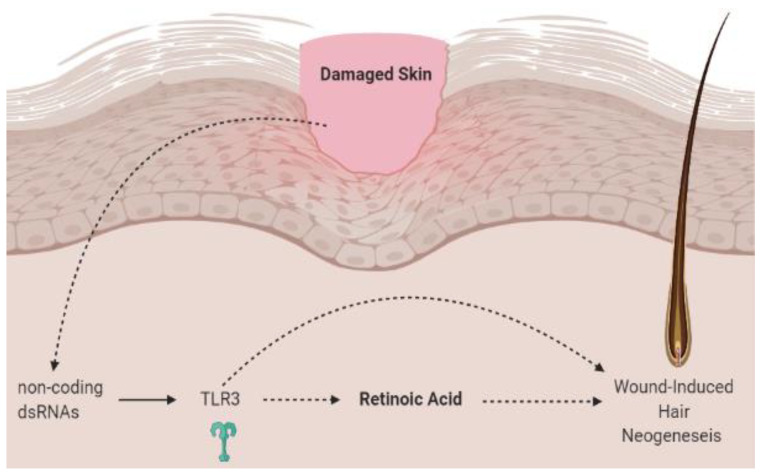
Wound-induced hair neogenesis. Skin injury stimulates non-coding dsRNA to activate TLR3 signaling, which triggers retinoic acid and mediates wound-induced hair neogenesis. Created with BioRender. Adapted from Kim et al. [21]. dsRNA, double-stranded RNA; TLR3, toll-like receptor 3.

In the skin, TLR2 and TLR3 biology is dependent on retinoic acid [11,12]. The ligand for TLR3 is double-stranded RNA (dsRNA). In skin, dsRNA that can activate TLR3 is generated by both skin damage and live viruses [13,14]. It has previously been shown that damaged skin activates TLR3 and induces follicular regeneration through a process known as wound-induced hair neogenesis (WIHN) [12,13,15,16] (Figure 2). Emerging evidence suggests that WIHN requires both TLR3 and RA [12,17]. TLR3 induces RA synthesis and signaling, which in turn promotes follicular hair regeneration [12]. Kim et al. show in mice and human keratinocytes that dsRNA induces intrinsic RA synthesis in a TLR3-dependent manner [12]. Thus, removal of TLR3 in the *Tlr3*^−/−^ mouse model abrogates RA production and hair follicle regeneration [12]. The observation that TLR3 triggers RA synthesis and signaling to promote regeneration also implies that combining TLR3 agonists with RA could have therapeutic benefits in skin and hair regeneration.

TLR2 is also impacted by RA. Di- and tri- acylated lipoproteins—often found on Gram-negative and Gram-positive bacteria—act as ligands to TLR2 [18]. TLR2-related pathways are essential for triggering the antimicrobial activity of keratinocytes against staphylococcal infections [18,19,20]. Liu et al. show in primary human monocytes that treatment with all-trans retinoic acid (ATRA) down-regulates TLR2 and its co-receptor, CD14 [11]. Additionally, treatment of the monocytes with ATRA decreased TLR2-induced release of the proinflammatory cytokine, IL-6, from CD14^+^ monocytes by 74 percent [11]. These data suggest that agents such as ATRA that target TLR expression and function may provide new strategies for treating inflammatory dermatologic conditions.

### 2.2. Retinoids and Keratinocytes

Keratinocytes are epithelial cells of the skin that express PRRs that activate the immune response when exposed to pathogens [22]. The downstream signaling that results from the interaction between PRR and pathogen-association molecular patterns (PAMPs) leads to the release of proinflammatory cytokines that modulate the immune response [22,23]. It has been recognized that both vitamin A deficiency and excess can lead to abnormal epithelial keratinization [24,25,26] as seen in acne vulgaris and psoriasis [27,28]. There is limited understanding of the mechanism by which retinoids regulate keratinocyte cell proliferation. A few in vitro and in vivo studies have shown that retinoids modulate epidermal proliferation by exerting an anti-proliferative effect on keratinocytes [23,29] through modulation of keratinocyte differentiation [30,31], tyrosine kinase expression [26,32] and posttranslational processing of keratinocyte proteins [33]. As there is no unified understanding that explains the effect of RA on keratinocyte development, a multicomponent mechanism may best explain the therapeutic success of retinoids in treatment of hyperkeratotic skin conditions. 

### 2.3. Retinoic Acid and Dendritic Cells

Dendritic cells are APCs that recognize external antigens and present them to naïve T cells. These cells are present in three subtypes: dermal dendritic cells, plasmacytoid dendritic cells and langerhans cells, which are the main DC subset in the epidermis [34]. Langerhans cells (LCs) are among the first dendritic cells to encounter microbial antigens [34]. In previous years, retinoids were found to contribute to LC maturation [35,36]. In an early study, isolation of LC from BALB/c mice treated with topical all-trans-retinoic acid, with or without ultra-violet radiation, induced an enhanced mixed epidermal cell lymphocyte reaction and enhanced allogeneic immune response [35]. A more recent study found that the development of LCs and langerin^+^ conventional DCs, found in the dermis and other tissues, is regulated by RARα in a RA-concentration-dependent manner [37]. Mice deficient in the RARα, due to deletion of the Rara gene, had defective LC development and failed to give rise to LCs in the skin [37]. The in vitro studies in both mice and human cells suggest that environmental influences on the RA-RARα axis regulate LC development [37]. In hypo-RA conditions, RARα promoted development of DC populations, and this was inhibited by rises in the systemic concentrations of RA [37]. The modulation of LC function by RA in these studies may explain the mechanism behind the treatment of dermatologic conditions with retinoids. However, the current knowledge on the direct effect of retinoids on DCs in the treatment of dermatologic conditions such as psoriasis is sparse and remains controversial [38,39]. This may be attributable to the complex involvement of various stimuli such as cytokines and ligands for pattern recognition receptors on the differentiation of DCs and variable effects on RAR types [3,40,41]. 

Notably, a recent investigation in a mouse model of psoriasis showed that activation of the retinoic-acid inducible-gene I (RIG-1), one of the major sensors of RNA viruses, triggers psoriasis-like skin disease through mediating endogenous IL-23 production by CD11c^+^ dendritic cells [42]. These findings suggest that in genetically predisposed individuals, viral infection may trigger psoriasis through the activation of TLR7, TLR8 and/or RIG-1 antiviral signaling and induction of IL-23 expression in CD11c^+^ dendritic cells [42]. IL-23 production in dendritic cells is NFκB-dependent. Therefore, in the setting of uncontrolled IL-23 release, mutations in NFκB may impair negative regulation of its proinflammatory activity leading to psoriasis [42]. This model warrants further consideration in the context of our current understanding of the role of vitamin A on immunity, as RIG-1 expression is induced by retinoic acid. Further, deficiencies in vitamin A have been shown to compromise responses to infection and vaccination [7]. Perhaps this hampered anti-viral response in vitamin-A deficiency is partially due to deficiency in RIG-1 signaling leading to a decreased T-helper 17 (Th-17) response. Indeed, there is diminished T-helper 17 (Th-17) response observed in vivo [7] in the setting of vitamin A deficiency. Th-17 cells are elicited via the actions of multiple cytokines, including IL-23, which is produced by DCs. Thus, disturbances to the regulation of DCs by RA and RIG-1 may impart changes that modulate the adaptive immune response. Physiologic concentrations of retinoids appear to sustain Th-17 development and maintenance [7,43,44]. Thus, it may be the case that enhanced activation of RIG-I due to NF-κB mutations disrupts skin homeostasis and gives rise to a hyperactive innate immune response, leading to the inflammation seen in psoriasis. 

### 2.4. Vitamin A and Mast Cells 

Vitamin A and its derivatives also play a role in mast cell regulation. Connective tissue mast cells are constitutively rich in the skin [45,46]. These innate immune cells increase in number and are functionally altered in atopic dermatitis [47], psoriasis [48] and chronic urticaria [49]. These cells express high affinity IgE receptor (FceRI) and TLRs (1–4, 6, 7 and 9) that respond to various microbial products, resulting in secretion of proinflammatory cytokines in the immune response [50]. RA is commonly used for treatment of inflammatory skin conditions and may have a complex role in mast cell regulation. It has been shown that all-trans RA can act on RARα in mast cells, resulting in the release of cytokines such as IL-1β, tumor necrosis factor-α (TNF α) and IL-8 from mast cells [51]. As shown in in vitro and in vivo studies in mice, this mechanism may be due to RA activation of Mas-related G-protein-coupled receptor-X2 (MRGPRX2), which induces mast cell degranulation, enhancing innate immunity against bacteria [52]. Since mast cells interact with T cells and are modulated by cytokines released from these adaptive cells [50], RA may modulate mast cell activity via both innate and adaptive immune processes. Vitamin A deficiency has also been shown to exacerbate extrinsic atopic dermatitis due to augmentation of Th2-mediated inflammation and mast cell activation, further supporting vitamin A as a regulator of mast cell activity [27].

## 3. Vitamin A, Antimicrobial Proteins and the Skin Microbiome

The epithelium of the skin, like that of the intestine, interfaces directly with the external environment and encounters a diversity of pathogens including bacteria, fungi and viruses [53,54,55]. Keratinocytes are pro-inflammatory cells of the epidermal layer of the skin that produce antimicrobial proteins (AMPs). AMPs belong to an array of protein families that function to rapidly kill or inactivate micro-organisms [56]. Epithelial AMPs play an essential role in preventing pathogenic invasion of the skin [56]. Amongst the several distinct AMP families that have been identified in the skin, cathelicidins, S100 proteins and β-defensins have been described [56]. Emerging research has sought to identify the role of vitamin A in AMP expression in the skin and the promotion of innate immunity against infection [2]. 

### 3.1. Vitamin A and Resistin-Like Molecule α 

**Figure 3:**
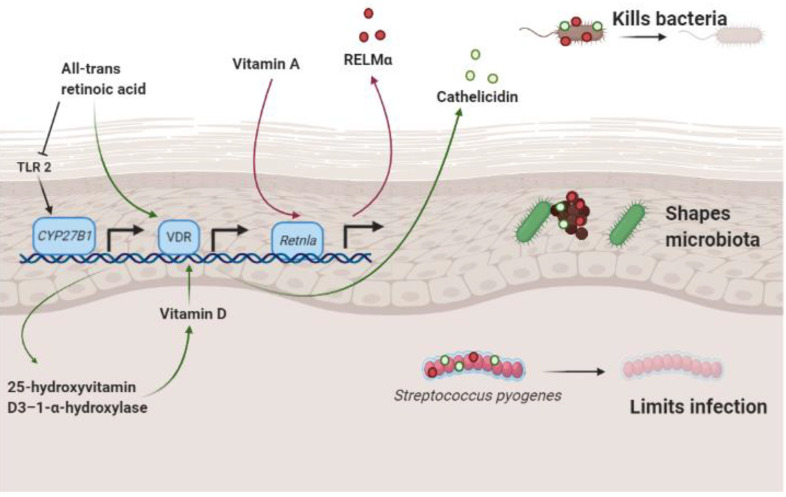
Schematic diagram of RELMα and cathelicidin involvement in skin immunity and the skin microbiome. Dietary vitamin A triggers the transcription of the Retnla gene which encodes the RELMα antimicrobial protein. When expressed, RELMα kills bacterial species that colonize the skin, shapes the resident microbiota and reduces the viability of invading pathogens such as *Streptococcus pyogenes* to limit infection. All-trans retinoic acid stimulates transcription of cathelicidin. When expressed, cathelicidin potentiates the immune response against microbial infection. Created in BioRender; adapted from Harris et al. [2]. RELMα, Resistin-like molecule α; Retnla, Resistin-like alpha precursor; CYP, cytochrome P450 family; VDR, Vitamin D receptor; TLR, toll-like receptor.

Recent work has established that the Resistin family of proteins has anti-microbial properties [2,57]. Mouse skin expresses Resistin-like molecule α (RELMα) and Resistin. Human skin expresses the protein Resistin. Both mouse RELMα and human Resistin kill bacteria at low micromolar concentrations in skin (Figure 3). Retnla^−/−^ mice, which do not express RELMα, are more susceptible to skin infection and have an altered skin microbiome. Interestingly, dietary vitamin A is required for RELMα expression in mice and mice treated with the vitamin A analog, isotretinoin, had increased expression of RELMα and increased resistance to infection of the skin [2]. Thus, dietary vitamin A plays an important function in regulating skin immunity. 

### 3.2. Retinoic Acid and Cathelicidin 

Cathelicidin is an AMP that is expressed by keratinocytes, sebocytes and adipocytes in skin. In a recent study, retinoic acid enhanced cathelicidin expression in adipocytes during reactive adipogenesis in vitro and in vivo in mouse and human preadipocytes [58]. During reactive adipogenesis, dermal fibroblasts of the preadipocyte lineage undergo rapid, local proliferation and differentiation in response to bacterial infection of the skin [59]. This process is important in skin innate immunity as it increases the expression of cathelicidin, which enhances the immune response against bacterial infection [60] (Figure 3). Given that retinoids typically inhibit adipogenesis, the enhancement of cathelicidin expression in preadipocytes treated with RA is suggestive of a more complex immunomodulatory role of RA in the skin and the immune defense. 

### 3.3. Vitamin A and Staphylococcus aureus 

Prior studies have shown that vitamin A deficiency leads to a state of immunodeficiency [61]. This immunodeficient state may predispose subjects to an increased incidence of *Staphylococcus aureus* (*S. aureus*) skin infection [61,62,63]. There is growing evidence that *S. aureus* colonization [63,64,65] and vitamin A deficiency [27,61,66,67] are influential in atopic dermatitis disease pathogenesis. Subjects with atopic dermatitis have increased bacterial colonization with *S. aureus* due to a multitude of factors, including a defective innate immune response [68], and this finding correlates with their notably less diverse skin microbiome [62,69]. These interesting observations encourage further exploration of the regulatory role of vitamin A and its derivatives on skin immunity, the skin microbiome and the development of inflammatory skin disease.

### 3.4. Vitamin A, Retinoic Acid and the Hair Microbiome

It is well established that RA has an important role in hair growth, differentiation and regeneration [70,71,72]. Much less is known about the interplay of RA, hair immunity and the hair microbiome. Within the skin, the hair follicle serves as a microbial niche for bacteria [73], fungi [74] and viruses [75]. Below the bulge region of the hair follicle, the epithelium is an immune-privileged territory that is restricted from the antimicrobial activity of immune cells [76,77,78]. Given its immune privilege, it is possible that the hair follicle employs the actions of AMPs such as cathelicidin to maintain homeostasis of the hair microbiome [79]. Emilianov et al. show that lesional skin from patients with hidradenitis suppurativa has an increased abundance of cathelicidin in the distal outer root sheath of the affected hair follicle [79]. Given the inducing effects of RA on cathelicidin in the skin during reactive adipogenesis [58], these data suggest a similar mechanism may regulate the microbiota environment of the hair follicle. As the human hair follicle microbiome remains to be fully characterized, current understanding of the hair microbiome is best extrapolated from evidence of the skin microbiome. Thus, the role of vitamin A in the maintenance of the human hair microbiome is a challenging question that requires further primary investigations. 

## 4. Conclusions

In this review, we summarize the effect of vitamin A, RA and retinoids in immune processes that promote skin immunity. These processes are intricately involved in the homeostasis of the skin microbiome and may be extrapolated to processes that regulate hair follicle immunity. Understanding the role of vitamin A and its derivatives on the innate immune system and the skin microbiome is of particular significance to translational human skin biology research and the development of therapeutic targets. Generating robust data on the mechanistic interplay of vitamin A and its derivatives on skin immunity and the skin microbiome may inform disease outcome and treatment response to agents that modulate innate immune signaling and AMP production. 

## Data Availability

Not applicable.
